# 

**Published:** 1873-06

**Authors:** 


### CONTENTS.

ORIGINAL COMMUNICATIONS.

Vegetable Fungi Growing in the Human Ear—Aspergillus Nigricans or Mykomyringitis. By A. R. Kilpatrick, M.D., . . . . . . . 321

A Dichotomous Analysis of Diseases Founded on their Symptoms. By W. T. Grant, M.D., . . . . . . . . . . . . . 325

Medical Extracts—Practical and Philosophical, No. II. By L. B. Anderson, M.D., . . . . . . . . . . . . . . 341

EXTRACTS AND GLEANINGS.

Treating Opacities of the Cornea—343. Arsenic in Diseases of the Liver—344. Cancer and Carbolic Acid—345. Remedy for Tapeworm—346. The Treatment of Goitre—348. Transmission of Syphilis—349. On the Pulvis Glycyrrhizæ Compositus, a Laxative Preparation of the Prussian Pharmacopœia—350. Hypodermic Use of Veratrum in Meningitis—351. Fibroid Tumors of the Uterus—352. Catarrh of the Nose—353. Treatment of Whitlow—354. Veratrum Viridi—355. Removal of Portions of Diseased Tissues in Cases of Carcinoma Uteri—356. The Treatment of Sciatica—357. An Improved Method of Taxis in Strangulated Hernia—359. Aural Affections in Children; On Temperature in Phthisis—360. Treatment of Erythema; Chloroform in Labor—361. Baptisia Tinctoria in Typhoid Fever; Phytolacca in Mastitis—362. Local Uses of Tannin—Purulent Ophthalmia Treated by Alcohol—363. Local Pain in the Right Side in Pregnancy; Strychnia in Typhoid Fever; Incontinence of Urine—364. Apparent Death; Delivering the Placenta; Atropia Poisoning—365. Effects of Compressed Air; The Influence of Bromide of Ammonium on Quinia; Dr. Hamilton's Means of Arresting Hemorrhage after Excision of Tonsils—366. Spasmodic Asthma; Uterine Hemorrhage; Reid's Cholera Sirup—367. Prevention of Mammary Abscess; Hepatic Abscess Cured by Injections of Iodized Iodide of Potassium; Formula for Constitutional Syphilis—368. Induction of Premature Labor; Iodine; Compound Arsenical Paper—369. Abortive Treatment of Boils and Felons; Pain in the Bladder or Penis; Hyoscyamia; Impotency—370. Treatment of Anthrax by Subcutaneous Aspiration; Iodine in Chronic Diarrhœa; Nitric Acid for Hemorrhoids; Belladonna in Intestinal Invagination and Hernia; An Application to Corns—371. The Natural Cause of Disease; Operation for Fistula in Ano; Salve for the Lips, etc.; For Excessive Perspiration of Hands or Feet; Ranula Cured by Galvano-Acupuncture—372. Squire's Vertebrated Prostatic Catheter; Bromide of Potassium in Epilepsy; How to Dissolve Alum in Bread; Vicarious Menstruation; Immediate Remedy for Hemoptysis—373.

EDITORIAL AND MISCELLANEOUS.

Ponce de Leon Spring, . . . . . . . . . . . . . 374
Communications; Medical Society of North Carolina, . . . . . 375
Toner Lectures; Abingdon Academy of Medicine, . . . . . . 376
Replies to Queries; Kind Words and Suggestions, . . . . . . 377
List of Cash Payments to the Record, . . . . . . . . . 379
Bibliographical, . . . . . . . . . . . . . . 380

